# Chemical Composition of Defatted Cottonseed and Soy Meal Products

**DOI:** 10.1371/journal.pone.0129933

**Published:** 2015-06-16

**Authors:** Zhongqi He, Hailin Zhang, Dan C. Olk

**Affiliations:** 1 USDA-ARS, Southern Regional Research Center, New Orleans, Louisiana, United States of America; 2 Dept. of Plant and Soil Sciences, Oklahoma State University, Stillwater, Oklahoma, United States of America; 3 USDA-ARS, National Laboratory for Agriculture and the Environment, Ames, Iowa, United States of America; United States Department of Agriculture, UNITED STATES

## Abstract

Chemical composition is critical information for product quality and exploration of new use. Hence defatted cottonseed meals from both glanded (with gossypol) and glandless (without gossypol) cotton seeds were separated into water soluble and insoluble fractions, or water soluble, alkali soluble as well as total protein isolates. The contents of gossypol, total protein and amino acids, fiber and carbohydrates, and selected macro and trace elements in these products were determined and compared with each other and with those of soy meal products. Data reported in this work improved our understanding on the chemical composition of different cottonseed meal products that is helpful for more economical utilization of these products. These data would also provide a basic reference for product standards and quality control when the production of the cottonseed meal products comes to pilot and industrial scales.

## Introduction

Cotton (*Gossypium hirsutum* L.) is produced in more than 30 countries [1] and provides a major fiber source for the textile industry. Much of the cotton land in the US is located in the southern and southeastern region which includes Georgia, Alabama, Arkansas, North Carolina, Mississippi, and Texas [2, 3]. Harvesting and ginning a cotton crop generates two marketable products: lint and seed. Although accounting for about 60% of biomass of cotton bolls, cottonseed products provide only a secondary revenue stream of the cotton crop (15–25% of the value of the crop), and mainly from the oil fraction [4, 5]. The residual fraction after oil crushing, called defatted cottonseed meal, is mainly used as fertilizers or animal feed [2, 5–9]. Thus, enhanced utilization of meal products as industrial and biobased raw materials would increase the profitability of cotton growers and processors. The potential value-added products include but are not limited to wood adhesives [10], bioplastics and films [11], superabsorbent hydrogel [12] antioxidant meal hydrolysates [13], as well as bio-oil and biochar [14]. These functional products differentially utilize the chemical components (e.g., proteins, peptides, and carbohydrates) in meal although expensive purified fractions are not always necessary.

For better and economic utilization of the different functional fractions in cottonseed meal, we have separated meal into a water soluble fraction (WSF) and a water insoluble fraction (WIF) [15]. Further work demonstrated that WIF could serve as bio-based wood adhesives [16, 17]. The protein fraction in meal can be extracted by weak base as a whole cottonseed protein isolate (PI), or sequentially extracted into a water soluble protein fraction (PIw) and an alkali soluble protein fraction (PIa) [18, 19]. Fluorescence study has shown that PIw is more hydrophilic than PIa [20]. This observation suggests PIw and PIa may not always behave the same when they are used to prepare a functional product. For example, Nordqvist et al. [21] reported that the wood adhesive per aqueous ethanol (60–70%) soluble gliadin fraction of wheat gluten penetrates to a larger extent and more deeply into the wood material than the adhesive per aqueous ethanol insoluble glutenin fraction of wheat gluten.

As chemical composition is critical information for product quality and for exploration of new uses, in this work, we determined the contents of gossypol, amino acids, fiber and carbohydrates, and selected macro and trace elements in these cottonseed meal fractions. For comparison, these parameters in defatted soy meal and its protein isolate were also determined, as soy meal is widely studied as a bio-based raw material [22–24]. Information derived from this work will be helpful in promoting enhanced utilization of these defatted oilseed meal fractions as renewable and environment-friendly industrial resources.

## Materials and Methods

### Raw materials

Meals from both glanded (with gossypol, Gd)) and glandless (without gossypol, Gl) cottonseeds were used. Gossypol [1,1,6,6,7,7-hexahydroxy-5,5-diisopropyl-3,3-dimethyl-(2,2-binaphthalene)-8,8-dicarbaldehyde, or 2,2′-bis-(formyl-1,6,7-trihydroxy-5-isopropyl-3-methylnaphthalene)] is a yellow polyphenolic binaphthyl dialdehyde stored in the pigment glands of cotton and a few related species [5, 25]. Three glanded samples came from three expander-solvent processers. Two glandless meal samples were donated by Cotton, Inc. (Cary, NC, USA) and received as partially defatted products. It was re-extracted with hexane at 50°C for 2 h in a rotary evaporator, which was sufficient to reduce the oil content to less than 1%.

Soy meal was obtained from Kentwood Co-op (Kentwood, LA, USA). The working soy meal was obtained by grinding the meal in a cyclone sample mill (Model 3010–014, UDY Corporation, Fort Collins, CO, USA) to pass a 0.5-mm steel screen [26].

### Preparation of water washed meals and protein fractions

The sequential fractionation procedure reported in He et al. [15] was used to separate the whole meal into water soluble and insoluble fractions of both cottonseed and soybean meals. Total protein isolates (PI) of cottonseed and soy meals were prepared by one-step alkali extraction and acid precipitation. PIw and PIa fractions of Gd cottonseed protein were sequentially extracted by water and 0.015 M NaOH, and then precipitated at pH 4.0 and 7.0, respectively [18]. Both fractions were freeze dried and kept in a dessicator at room temperature (22°C) until use.

### Gossypol in cottonseed samples

The gossypol enantiomers in Gd meal and products were detected by a slightly modified procedure based on AOCS Recommended Practice Ba 8a-99 using about 100 mg sample for each analysis [4, 27]. High pressure liquid chromatography was used to detect (+)- and (-)-gossypol after the compounds were extracted and transformed into Schiff’s base derivatives with *R*-(–)-2-amino-1-propanol. The gossypol complex was detected at 254 nm. A standard curve was constructed for each isomer with serial dilutions of racemic gossypol-acetic acid (1:1) in complexing reagent. Total gossypol was calculated from the sum of the individual (+)- and (–)-gossypol isomers. Percent (+) gossypol represents the amount of the (+) isomer divided by the sum of the (+) and (–) isomers, expressed as a percentage.

### Determination of total N, crude protein, and amino acids

The concentrations of total N in each sample were determined using a LECO Truspec dry combustion Carbon/Nitrogen Analyzer. Crude protein content in the samples were calculated by multiplying the total N by a factor of 6.25 [7]. Ion chromatography coupled with amperometric detection was used to measure 17 proteinous amino acids(AAs) and four nonproteinous amino compounds (2 amino acids and 2 amino sugars, for convenience hereinafter also abbreviated as AAs) in the cottonseed [7, 28]. A proteinous AA (tryptophan) was not measured by this method [29, 30]. Each sample (20 mg) was mixed with 2 mL of 4 M methanesulfonic acid (MSA) amended with 2 g L^-1^ tryptamine and autoclaved for 16 h at 121°C (208 kPa). The acid extracts were titrated to pH 4 to 5 with NaOH and centrifuged to remove precipitates. The aliquots were diluted properly with purified water. Concentrations of amino acids in these diluted solutions were analyzed by a Dionex DX-500 (Dionex Corp. Sunnyvale, CA) ion chromatograph equipped with an Amino-Pac PA 10 column (2 mm i.d.). Triple pulsed amperometrric detection was performed using a Dionex ED-40 electrochemical detector. As the concentration (mg mL^-1^) of individual AA was used for the standard curve, a conversion factor {i. e. [molecular weight-18(water)]/ molecular weight}was used to calculate each AA's content in protein peptides from the measured free AA content [31].

### Determination of dietary fibers and carbohydrates

Acid detergent fiber (ADF), neutral detergent fiber (NDF) and acid detergent lignin (ADL) were determined using the filter bag methods with an Ankom Fiber Analyzer (Ankom Technology, Macedon, NY) [7].

Seven carbohydrates (fucose, arabinose, rhamnose, galactose, glucose, xylose, and mannose) were extracted and analyzed per literature [32–34]. Briefly, 800 μL of 6M H_2_SO_4_ was added to 100 mg of a sample. After mixing, the solution was allowed to sit for 30 minutes at room temperature then diluted to 1M H_2_SO_4_. This solution was autoclaved for 30 min at 121°C, then centrifuged and the supernatant removed following quantitative rinsing of the remaining residue using distilled water. This residue was saved and sequentially processed for strong-acid extractable carbohydrates as described below. The supernatant containing the mild-extractable carbohydrates was pH adjusted with NaOH to 5.5–6.5, diluted, and an aliquot was injected into the Dionex DX-500 anion chromatograph equipped with a CarboPac PA-10 column (2 mm diameter x 250 mm length). All carbohydrates were detected with triple-pulsed amperometry. The stronger acid extraction, which isolated hydrolyzed glucose, was performed on the dried residue from the weaker acid extraction using 300 μL of 18M H_2_SO_4_ which was then diluted to 1.5 M after sitting for 30 min, followed by autoclaving, pH adjustment and dilution similar to the amino acid analysis.

### Elemental analysis

The element contents of the samples were analyzed following digestion (Jones and Case, 1990) in which 0.50 g of ground cottonseed sample was digested in 10.0 mL of concentrated trace metal grade HNO_3_ for one hour in the HotBlock Environmental Express block digester. The sample was then heated to 115°C for 2 h and 15 min. The concentrations of 12 elements (i. e. Al, B, Ca, Cu, Fe, K, Mg, Mn, Na, P, S, and Zn) in these digests were determined by a Spectro CirOs ICP spectrometer (Mahwah, NJ, USA) [35].

### Statistical analysi*s*

The data analysis package in Microsoft Excel 2007 was used for statistical analysis. The Descriptive Statistics Tool Data was used to calculate averages and standard errors. "Single-factor" analysis of variance (ANOVA) was used to evaluate the significance of the difference in amino acid composition between different meal fractions. The Correlation Analysis Tool was used to analyze correlation coefficients between the two sets of total protein data.

## Results and Discussion

### Distribution of gossypol enantiomers in glanded cottonseed meal products

The total content of gossypol was 6.82, 8.20, and 15.36 g kg^-1^ in the three meal samples with an average of 10.12 g kg^-1^ and standard error of 2.64 g kg^-1^ (Table 1). The high variation of the gossypol content apparently led to great standard errors in the separated fractions (e. g. PI and PIw). Previously, Berardi et al. [36] reported the gossypol content ranged from 2.5 to 13.5 g kg^-1^ in high and low gossypol cottonseed flours. Even though the content of total gossypol differed greatly among the meal samples, the ratio of the two enantiomers was basically unchanged with 60% as (+) enanmiomer in all three samples, similar to that of the whole cottonseed [4]

**Table 1:** Content of gossypol enantiomers in glanded cottonseed meal products.

	(+) Enantiomer	(-) Enantiomer	Total	Ratio of (+/-) Enantiomer
	g kg^-1^ of sample			
Meal	6.11 ± 1.61	4.01 ± 1.04	10.12 ± 2.64	1.51 ± 0.02
WIF	3.75 ± 0.05	2.45 ± 0.02	6.21± 0.04	1.53 ± 0.03
WSF	3.51 ± 0.01	2.54 ± 0.02	6.05 ± 0.02	1.38 ± 0.01
PI	3.16 ± 2.27	2.11 ± 1.45	5.27 ± 3.74	1.43 ± 0.04
PIw	5.27 ± 2.51	3.63 ± 1.61	8.91 ± 4.12	1.43 ± 0.06
PIa	3.38 ± 0.36	2.22 ± 0.25	5.60 ± 0.61	1.52 ± 0.01

Data are presented in average with standard error (n = 2 or 3).

**Fig 1:**
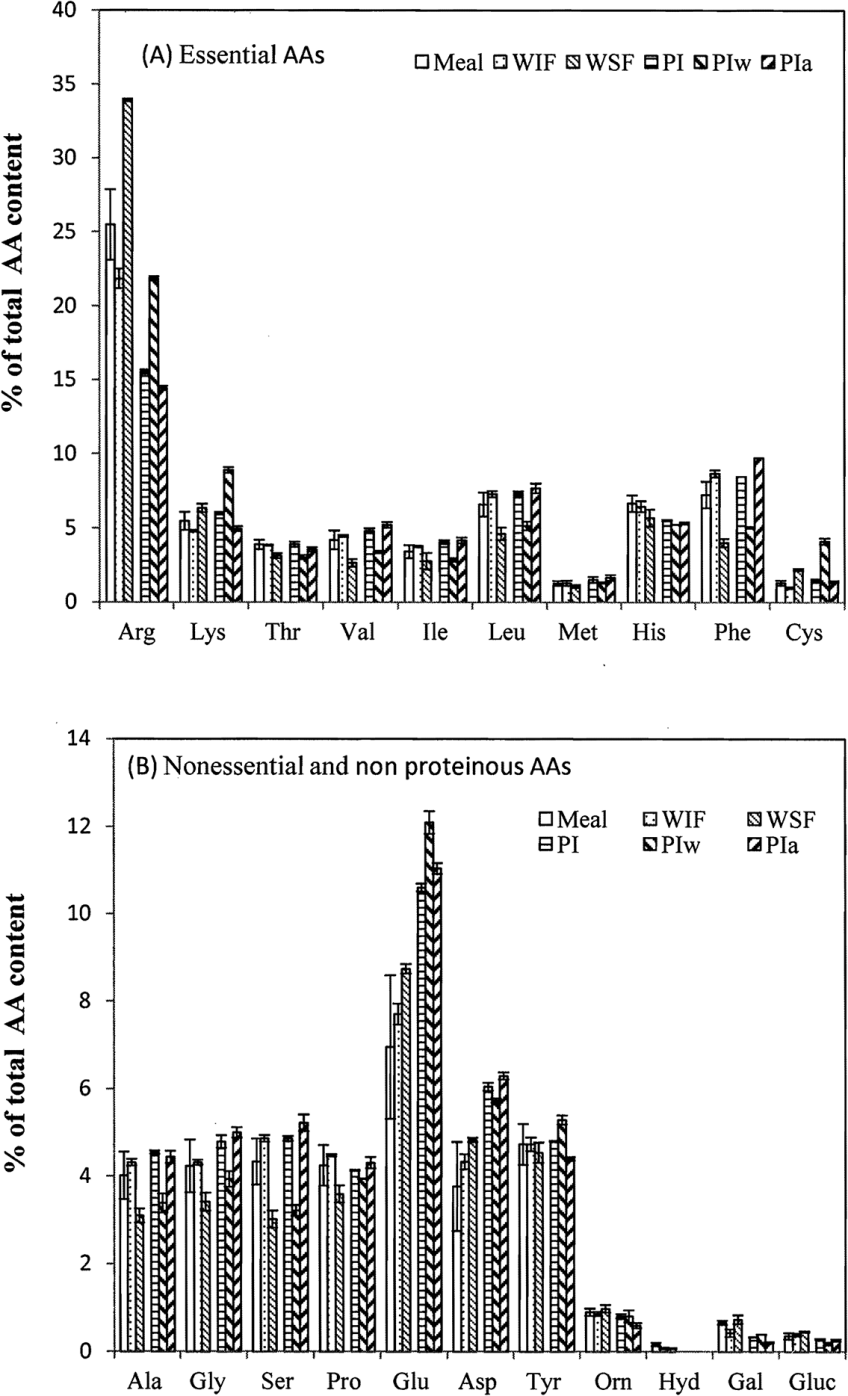
Contents of amino acids in glanded (Gd) cottonseed meal and their water soluble (WSF) and insoluble (WIF) fractions, total protein isolate (PI) and water (PIw)- and alkali (PIa)-extracted protein isolates. Data are presented in average with standard error (n = 2 or 3).

As shown in Table 1, when meal was separated into the water soluble and insoluble fractions, gossypol seemed equally distributed in the two fractions. However, gossypol content was higher in PIw than in PIa when the two protein fractions were sequentially extracted from the meal. Gossypol content of PI was similar to that of PIa, as the majority of PI was the PIa fraction [18]. Free gossypol in meal can become "bound" by reacting with the free ε-amino groups of lysine [37]. Hence the higher gossypol content in PIw is consistent with the higher lysine content in PIw than in PIa (Fig 1).

### Crude protein content and amino acid profiles

Estimated from total N contents, the contents of crude protein in glanded and glandless cottonseed meals and soy meal were 50.7, 59.4 and 49.6% of the dry matter, respectively (Table 2). Water washing enriched the protein content in the washed meals (i. e. the insoluble fraction WIF) by 12 to 25%, accompanied by a decrease in the protein content of WSF. The protein contents of both the PI and PIa of both cottonseed meals neared 100%, while soy protein isolate (SPI) contained about 92% protein. The protein content of PIw was 81%. These data were consistent with our previous reports [15, 18]. The protein contents calculated from the sum of AAs were all higher than the corresponding values calculated from total N. Yet both modes of protein content calculation provided for similar trends among the samples. As a matter of fact, As a matter of fact, the two sets of protein content data were highly correlated with the linear refression:% of AA-based content = 0.835* % of total N-based content +25.4 (r = 0.976, *P* < 0.001).

**Table 2:** Content (% of dry matter) of crude protein in cottonseed and soy meal and their products.

	Glanded cottonseed		Glandless cottonseed		Soy	
	TN-based	AA-based	TN-based	AA-based	TN-based	AA-based
Meal	50.7 ± 5.2	69.3 ± 8.0	59.4 ± 0.2	67.4 ± 3.1	49.6 ± 0.1	65.8 ± 1.7
WIF	74.5 ± 0.7	89.7 ± 2.3	72.3 ± 1.4	84.2 ± 2.3	66.2 ± 1.5	80.1 ± 2.9
WSF	39.8 ± 0.2	59.1 ± 1.9	47.7 ± 0.2	66.9 ± 1.7	ND ^a^	ND
PI	97.4 ± 2.5	108.5 ± 0.8	104.0 ± 0.1	ND	92.0 ± 0.2	106.6 ± 1.6
PIw	80.6 ± 0.3	97.8 ± 1.1	-^b^	-	-	-
PIa	104.0 ± 0.3	105.4 ± 2.7	-	-	-	-

Data are presented in average with standard error (n = 2 or 3) per total nitrogen (TN) and amino acid (AA) contents.

^a^ Not determined.

^b^ No PIw and PIa were prepared from glandless cottonseed and soy.

**Fig 2:**
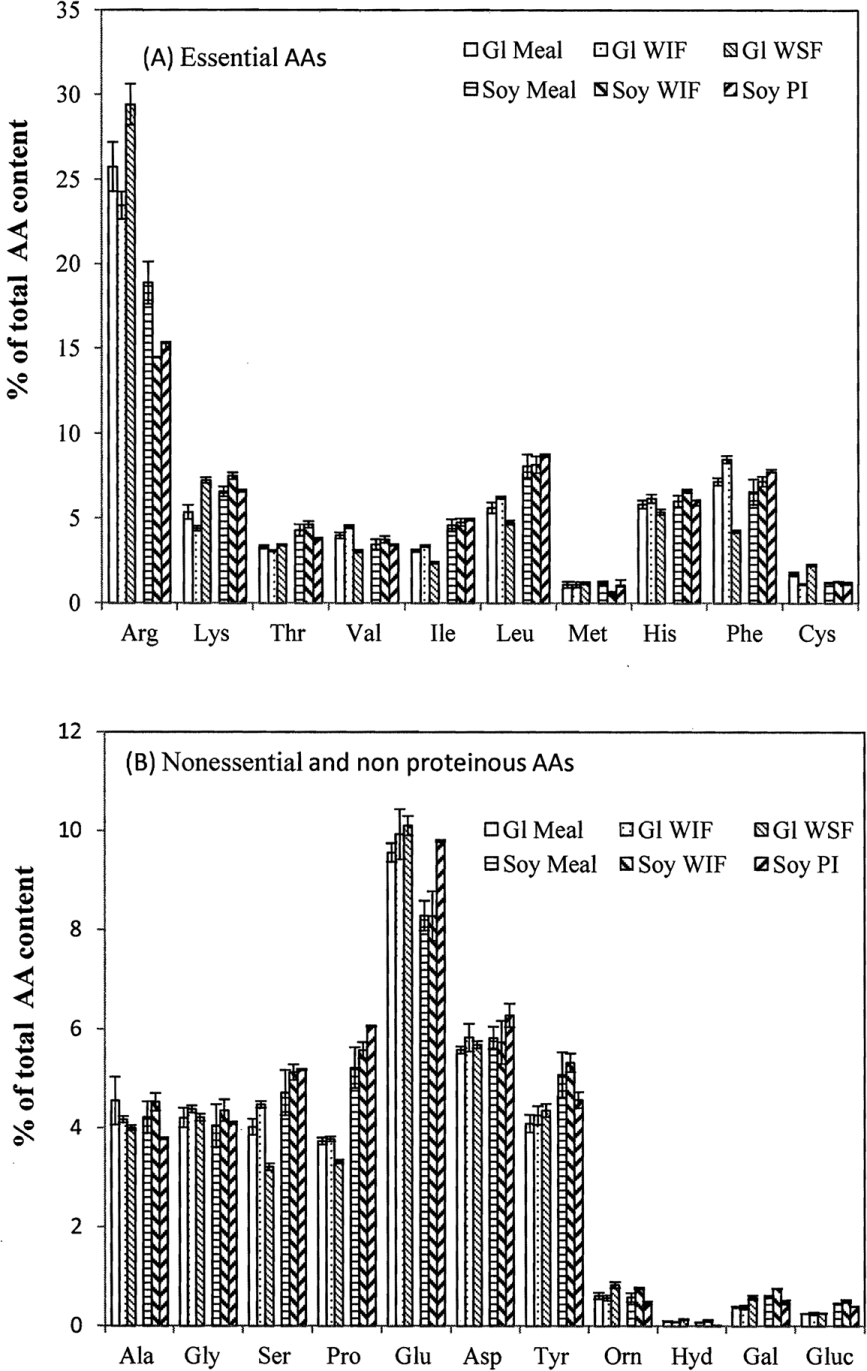
Contents of amino acids (AAs) in glandless (Gl) cottonseed and soy meal, and their water soluble (WSF) and insoluble (WIF) fractions, and total protein isolate (PI). Data are presented in average with standard error (n = 2 or 3).

The AA profiles of these samples are presented in Figs 1 and 2. Among the 10 essential AAs, the content of arginine was the highest, ranging from 15% to 34% of total protein (Fig 1). Many other essential AAs were around 5% of total protein with methionine and cystine having the lowest the lowest contents (1–2%). Glutamate (glutamine and glutamic acid) was the t most abundant (around 10% of total protein) of the seven nonessential proteinous AAs (Fig 2). The contents of the other six nonessential AAs ranged from 3% to 6% of total protein. None of the four nonproteinous AAs and sugars accounted for more than 1% of the total protein. Whereas there is no previous report on the contents of the four nonproteinous AAs in cottonseed and soy products, the relative abundances of most proteinous AAs in Figs 1 and 2 were similar to previous reports for whole cottonseed and defatted meal [38–40], except for arginine, which has been reported as the second highest after glutamate. One possible explanation for this discrepancy is the over-estimation of the arginine content in this work, as we had previously observed that the arginine peak, being the first eluted from the anion chromatographic column, could overlap with the void peak of residual seed oil in a cottonseed sample [7]. On the other hand, arginine was the most abundant amino acid in separated cottonseed protein peptides [41]. Among the five SDS-PAGE isolates of cottonseed protein bodies, two isolates (molecular weight 37.2–32.2 kD, and 21.9–18.0 kD) had higher arginine contents than all other amino acids, 26.5% and 24.4% of total protein for arginine vs. 17.6% and 17.7% for glutamate, respectively [41]. Thus, further research is needed to find out the true cause(s) of the highest arginine content in these samples.

Although there were some differences in the AA contents between the meal and their products, it is difficult to derive a general trend. For simplification, we grouped the total contents of 10 essential AAs, and 11 non essential proteinous and nonproteinous AAs (Table 3). Although the essential AA content did not always differ significantly (*P* > 0.05) among the fractions, it was generally greater in water soluble WSF fractions, and lower in the less soluble WIF, PIa and PI fractions, compared to the whole defatted meal. This observation may have some practical implications. As the WSF and PI of cottonseed meal are used as wood adhesives, the usefulness of the water soluble fractions toned deserves further exploration. At a minimum, these data imply the water soluble fractions of cottonseed meal are comparable to the whole meals regarding protein nutrient. In other words, these soluble fractions of cottonseed and soy meal can still be used as dairy cow protein supplement as the original CSM [6].

**Table 3:** Contents (percent of protein) of essential amino acids (EAAs), non-essential and non proteinous amino acids (NAAs), amino acids with polar side chains (AAsP), and amino acids with nonpolar side chains (AAsN) in glanded (Gd) and glandless (Gl) cottonseed and soy meals and their water insoluble (WIF) and soluble (WSF) fractions, total protein isolate (PI) and water(PIw)- and alkali (PIa)-extracted protein isolates.

	EAAs	NAAs	AAsP	AAsN
Gd Meal	65.8 a	34.2 a	62.7 a	35.2 a
Gd WIF	63.5 ab	36.5 ab	59.6 b	38.6 b
Gd WSF	66.5 a	33.5 a	72.5 ac	25.2 c
Gd PI	58.8 c	41.2 c	58.8 bd	39.7 bd
Gd PIw	61.0 ab	39.0 ab	69.5 c	29.1 c
Gd PIa	58.2 c	41.8 c	56.7 d	42.2 d
Gl Meal	62.9 a	37.1 a	65.2 a	33.4 a
Gl WIF	61.9 a	38.1 a	62.7 a	36.0 b
Gl WSF	63.3 a	36.7 a	71.0 b	27.1 c
Soy Meal	60.9 a	39.1 a	60.9 a	37.4 a
Soy WIF	59.0 b	41.0 b	59.0 a	38.9 b
Soy PI	58.9 b	41.9 b	58.8 a	39.0 ab

Data are presented in average (n = 2 or 3). Different letter after values in a column of the same type of meals indicate significantly difference at *P* ≤ 0.05.

We also regrouped these AAs as having either polar or nonpolar side chains (Table 3). The WSF and PIw contained more polar side chains than WIF and PIa, respectively. This observation explained our previous finding that PIa was less hydrophilic than PIw as revealed by fluorescence excitation-emission matrix spectroscopy [20]. Hettiarachchy et al. [42] reported that hydrophobicity plays an important role in wood adhesives. Thus, the better water resistance of the WIF-based adhesives than WSF [15] could be at least partially attributed to the more non polar (i. e. hydrophobic) AAs in the WIF.

### Contents of fiber and carbohydrates

Fiber components were present in both types of cottonseed meals in the order of NDF>ADF>ADL (Table 4). The order was the same as for the fiber components in the whole cottonseed [7, 38] even though the contents in the seed samples were about 3–5 times higher than in the meal samples. No fiber components were detected in WSF fractions, indicating their insolubility. Fiber components in the WIF fraction could be further subclassified as cellulose (ADF-ADL) or hemicellulose (NDF-ADF) fractions [43]. Thus, the WIF fractions of the two types of cottonseed meals contained 7.6% and 5.7% of dry matter as cellulose and about 0% and 3.6% as hemicelluloses, respectively.

**Table 4:** Contents (percent of dry matter) of acid detergent fiber (ADF), acid detergent lignin (ADL), and neutral detergent fiber (NDF) in glanded (Gd) and glandless (Gl) cottonseed meals and their water insoluble (WIF) and soluble (WSF) fractions.

	ADF	NDF	ADL
Gd meal	5.47 ± 1.48	11.58 ± 2.53	0.95± 0.81
Gd WIF	8.25 ± 0.92	8.24 ± 1.08	0.67 ± 0.17
Gd WSF	< DL ^a^	< DL	< DL
Gl meal	4.51 ± 0.37	7.88 ± 0.99	0.65 ± 0.60
Gl WIF	6.63 ± 0.58	10.21 ± 0.83	0.86 ± 0.74
Gl WSF	< DL	< DL	< DL

Data are presented in average with standard error (n = 2 or 3).

^a^ Less than detected limit.

**Fig 3:**
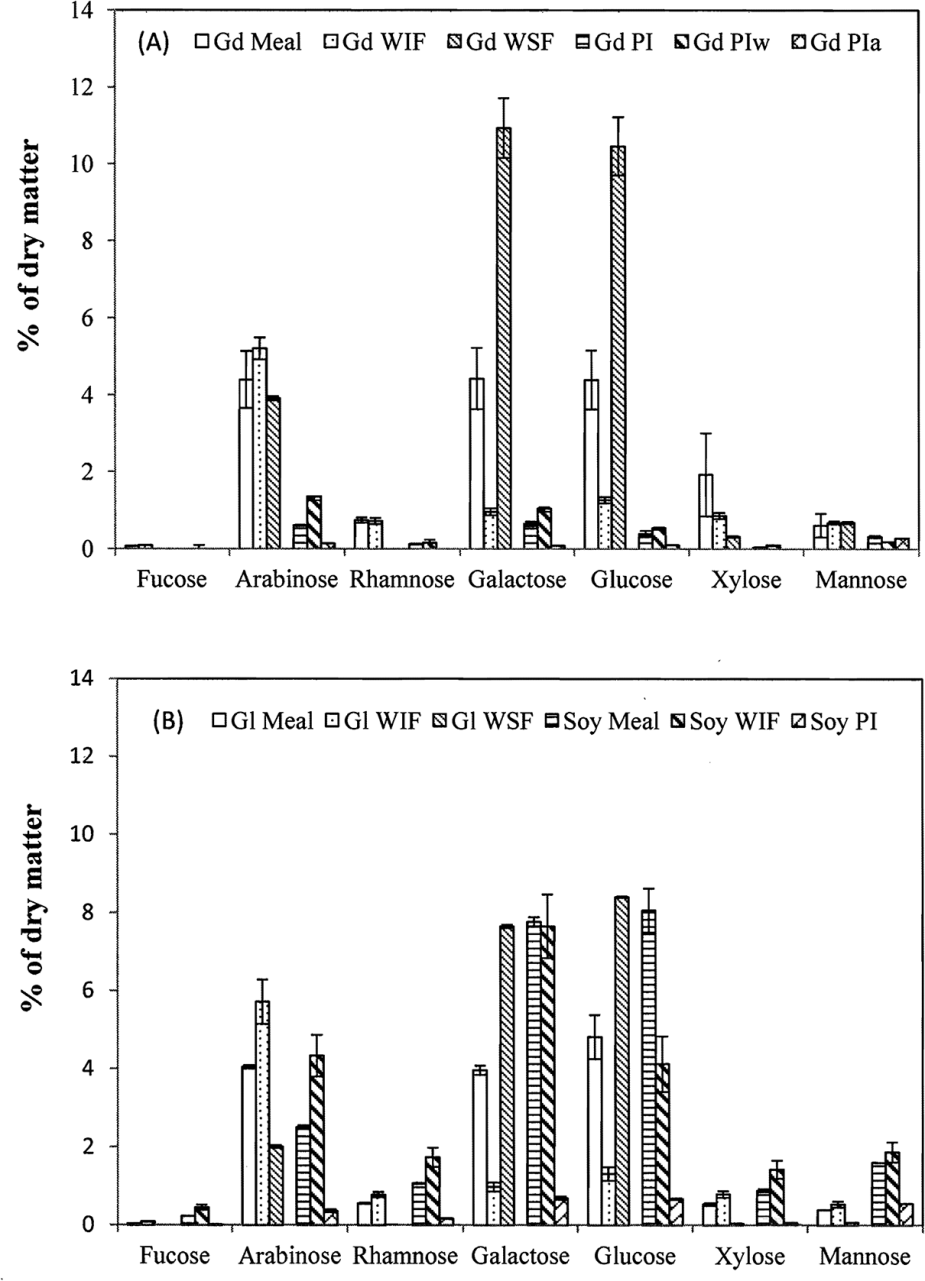
Selected carbohydrates in glanded (Gd) and glandless (Gl) cottonseed and soy meal, and their water soluble (WSF) and insoluble (WIF) fractions, total protein isolate (PI) and water (PIw)- and alkali (PIa)-extracted protein isolates. Data are presented in average with standard error (n = 2 or 3).

In addition to cellulose and hemicelluloses, seven other carbohydrates were measured in the meal and selected products (Fig 3). The contents of the carbohydrates in glanded cottonseed meal were in the order of galactose (4.43%) ≈ arabinose (4.40%) ≈ glucose (4.39%) > xylose (1.93%) > rhamnose (0.74%) ≈ mannose (0.61%) > fucose (0.07%), providing a total content of 16.6% of dry matter. The relative contents of these carbohydrates in the glandless meal were similar, with the total content of 14.4%. The relative contents of soy meal differed from the two cottonseed meals, with the order of glucose (8.06%) ≈ galactose (7.77%) > arabinose (2.51%) > mannose (1.61%) > rhamnose (1.07%) ≈ xylose (0.89) > fucose (0.23%) and a total content of 22.2% of dry matter.

The seven carbohydrates partitioned into WIF and WSF fractions differently. Galactose and glucose were mainly in the WSF fractions, excepting a high content of galactose in soy WIF. Generally, there was more arabinose, rhomonose, and xylose in the WIF fractions than in the WSF fractions of the three meals. The protein isolates also contained some of these carbohydrates. The distribution pattern of these carbohydrates in the Gd PIw and PIa protein fractions were similar to that of Gd WSF and WIF meal fractions although the carbohydrate contents in protein fractions were much lower. These carbohydrates were present perhaps as part of glycoproteins or impurity. In summary, the seven carbohydrates accounted for 16.6% and 14.4% of dry matter in the Gd and Gl meals, respectively. The total contents were 26.3% and 9.8% in the soluble and insoluble fractions of GD meal, and 18.2% and 10.2% of the same two fractions of Gl meal, respectively. The total contents in Gd protein isolate, water- and alkali-extracted fractions were 1.9%, 3.4%, and 0.6% of dry matter, respectively.

### Selected element contents

**Fig 4:**
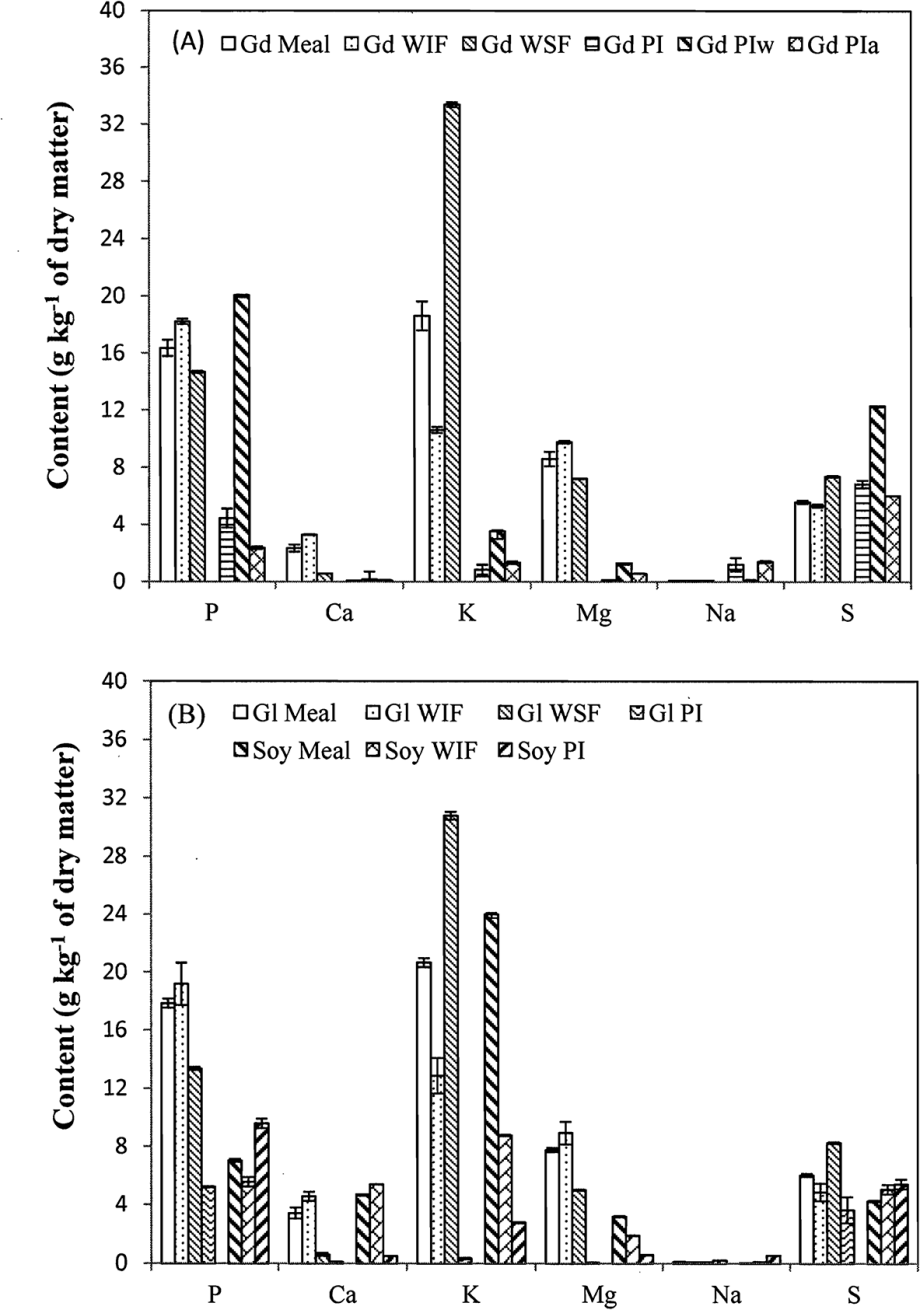
Contents of P, Ca, K, Mg, Na, and S in glanded (Gd) and glandless (Gl) cottonseed and soy meal, and their water soluble (WSF) and insoluble (WIF) fractions, total protein isolate (PI) and water (PIw)- and alkali (PIa)-extracted protein isolates. Data are presented in average with standard error (n = 2 or 3).

**Fig 5:**
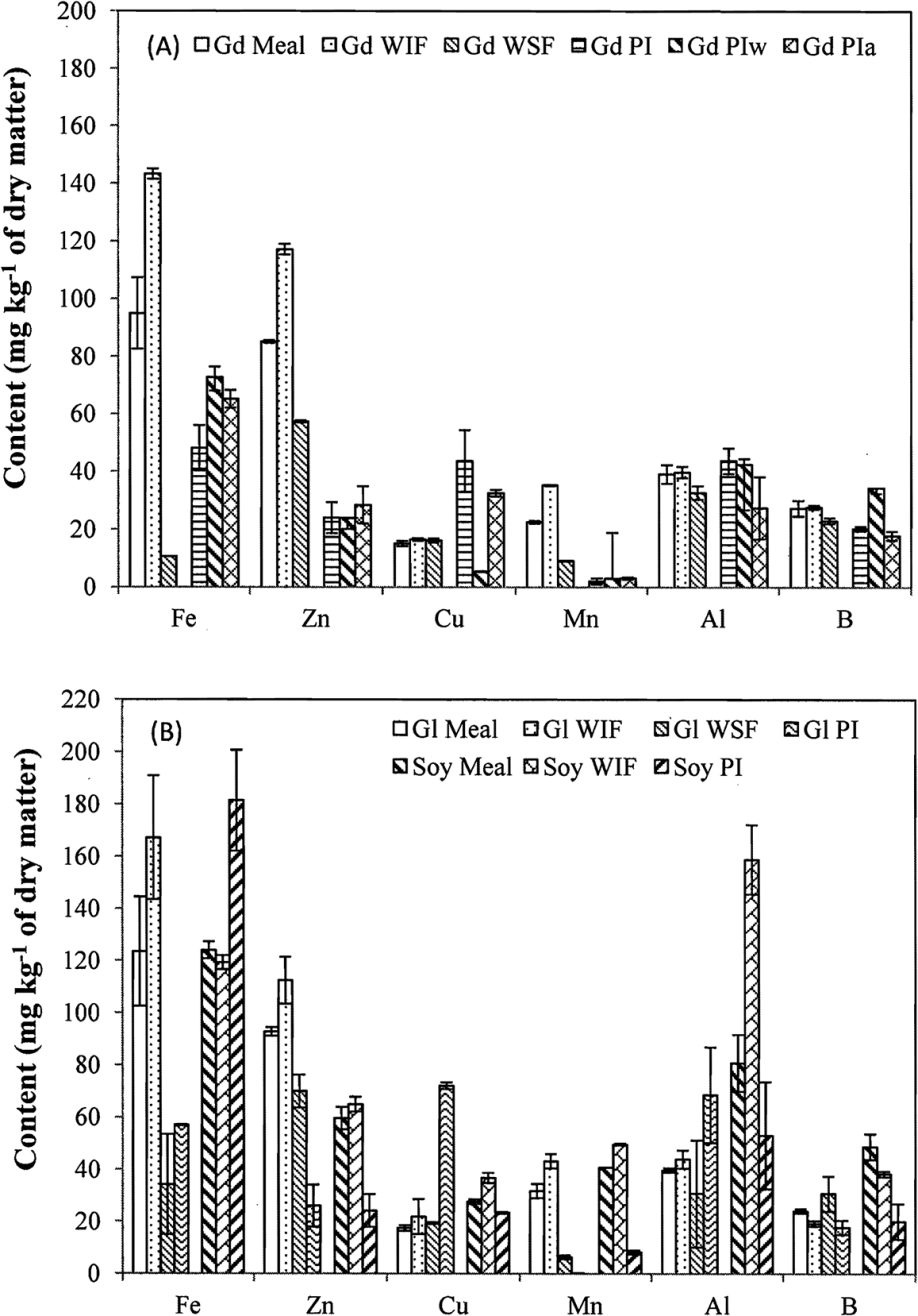
Contents of Fe, Zn, Cu, Mn, Al, and B in glanded (Gd) and glandless (Gl) cottonseed and soy meal, and their water soluble (WSF) and insoluble (WIF) fractions, total protein isolate (PI) and water (PIw)- and alkali (PIa)-extracted protein isolates. Data are presented in average with standard error (n = 2 or 3).

The contents of 12 selected elements are shown in Figs 4 and 5. Similar to the whole cottonseed [2, 35], the contents of macro elements P, Ca, K, Mg, Na, and S were generally higher than the six trace elements (Fe, Zn, Cu, Mn, Al, and B). Contents of either the macro- or micro-elements differed little between the two types of cottonseed meal. However, the contents of most elements in soy meal were lower than the corresponding elements in cottonseed meal. Compared to the meal samples, the contents of P, Ca, Mg, Fe, Zn, Mn, and Al increased in water insoluble fractions, but decreased in water soluble fractions, indicating the enrichments of these elements by water washing, likely due to the relative insolubility of polyvalent cations. In contrast, K and S had lower contents in the water insoluble fractions and higher contents in the water soluble fractions. In protein isolates and the water- or alkali-extracted fractions, only the contents of P and S were remarkably greater than their contents in meal samples. Sulfur is part of protein amino acids methionine and cysteine. Element P could be phytate (inositol phosphate) associated with seed proteins [44].

## References

[1] Campbell BT, Boykin D, Abdo Z, Meredith WR (2014) Cotton In: Smith S, Diers B, Specht J, Carver B (eds) Yield Gains in Major U.S. Field Crops. American Chemical Society, Madison, WI, p 13–32.

[2] Bellaloui N, Turley RB (2013) Effects of fuzzless cottonseed phenotype on cottonseed nutrient composition in near isogenic cotton (Gossypium hirsutum L.) mutant lines under well-watered and water stress conditions. Front. Plant Sci. 4:516 doi: 510.3389/fpls.2013.00516 24416037 10.3389/fpls.2013.00516 PMC3874854

[3] Tazisong IA, He Z, Senwo ZN (2013) Inorganic and enzymatically hydrolyzable organic phosphorus of Alabama Decatur silt loam soils cropped with upland cotton. Soil Sci. 178:231–239.

[4] Pettigrew WT, Dowd MK (2014) Nitrogen fertility and irrigation effects on cottonseed composition. J. Cotton Sci. 18:410–419.

[5] Dowd MK, Wakelyn PJ (2010) Cottonseed: current and future utilization In: Wakelyn PJ, Chaudhry R (eds) Cotton: Technology for the 21st. Century. ICAC Press, Washington D.C., p 437–460.

[6] Broderick GA, Kerkman TM, Sullivan HM, Dowd MK, Funk PA (2013) Effect of replacing soybean meal protein with protein from upland cottonseed, Pima cottonseed, or extruded Pima cottonseed on production of lactating dairy cows. J. Dairy Sci. 96:2374–2386. doi: 10.3168/jds.2012-5723 23462167 10.3168/jds.2012-5723

[7] He Z, Zhang H, Olk DC, Shankle M, Way TR, Tewolde H (2014) Protein and fiber profiles of cottonseed from upland cotton with different fertilizations. Modern Appl. Sci. 8(4):97–105.

[8] Li J, Li D, Zang J, Yang W, Zhang W, Zhang L (2012) Evaluation of energy digestibility and prediction of digestible and metabolizable energy from chemical composition of different cottonseed meal sources fed to growing pigs. Asian-Aust. J. Anim. Sci. 25:1430–1438. doi: 10.5713/ajas.2012.12201 25049499 10.5713/ajas.2012.12201 PMC4093011

[9] Wanapat M, Anantasook N, Rowlinson P, Pilajun R, Gunun P (2013) Effect of carbohydrate sources and levels of cotton seed meal in concentrate on feed intake, nutrient digestibility, rumen fermentation and microbial protein synthesis in young dairy bulls. Asian-Aus. J. Anim. Sci. 26:529–536. doi: 10.5713/ajas.2012.12607 25049819 10.5713/ajas.2012.12607 PMC4093391

[10] Cheng HN, Dowd MK, He Z (2013) Investigation of modified cottonseed protein adhesives for wood composites. Ind. Crop. Prod. 46:399–403.

[11] Yue H-B, Cui Y-D, Shuttleworth PS, Clark JH (2012) Preparation and characterization of bioplastics made from cottonseed protein. Green Chem. 14:2009–2016.

[12] Zhang B, Cui Y, Yin G, Li X, You Y (2010) Synthesis and swelling properties of hydrolyzed cottonseed protein composite superabsorbent hydrogel. Int. J. Polym. Mat. Polym. Biomat. 59:1018–1032.

[13] Gao D, Cao Y, Li H (2010) Antioxidant activity of peptide fractions derived from cottonseed protein hydrolysate. J. Sci. Food Agric. 90:1855–1860. doi: 10.1002/jsfa.4024 20602516 10.1002/jsfa.4024

[14] Singh V, Soni A, Kumar S, Singh R (2014) Characterization of liquid product obtained by pyrolysis of cottonseed de-oiled cake. J. Biobased Mat. Bioenerg. 8:338–343.

[15] He Z, Cheng HN, Chapital DC, Dowd MK (2014) Sequential fractionation of cottonseed meal to improve its wood adhesive properties. J. Am. Oil Chem. Soc. 91:151–158.

[16] He Z, Chapital DC, Cheng HN, Dowd MK (2014) Comparison of adhesive properties of water- and phosphate buffer-washed cottonseed meals with cottonseed protein isolate on maple and poplar veneers. Int. J. Adhes. Adhes. 50:102–106.

[17] He Z, Chapital DC, Cheng HN, Klasson KT, Olanya MO, Uknalis J (2014) Application of tung oil to improve adhesion strength and water resistance of cottonseed meal and protein adhesives on maple veneer. Ind. Crop. Prod. 61:398–402.

[18] He Z, Cao H, Cheng HN, Zou H, Hunt JF (2013) Effects of vigorous blending on yield and quality of protein isolates extracted from cottonseed and soy flours. Modern Appl. Sci. 7(10):79–88.

[19] Zhang B, Cui Y, Yin G, Li X, Zhou X (2009) Alkaline extraction method of cottonseed protein isolate. Modern Appl. Sci. 3(3):77–82.

[20] He Z, Uchimiya M, Cao H (2014) Intrinsic fluorescence excitation-emission matrix spectral features of cottonseed protein fractions and the effects of denaturants. J. Am. Oil Chem. Soc. 9:1489–1497.

[21] Nordqvist P, Thedjil D, Khosravi S, Lawther M, Malmstrom E, Khabbaz F (2012) Wheat gluten fractions as wood adhesives-glutenins versus gliadins. J. Appl. Polymer Sci. 123:1530–1538.

[22] Chen M, Chen Y, Zhou X, Lu B, He M, Sun S, Ling X (2014) Improving water resistance of soy-protein wood adhesive by using hydrophilic additives. BioResources 10:41–54.

[23] Qi G, Li N, Wang D, Sun XS (2013) Adhesion and physicochemical properties of soy protein modified by sodium bisulfite. J. Am. Oil Chem. Soc. 90:1917–1926.

[24] Tong X, Luo X, Li Y (2015) Development of blend films from soy meal protein and crude glycerol-based waterborne polyurethane. Ind. Crop. Prod. 67:11–17.

[25] He Z, Waldrip HM, Wang Y (2012) Application of capillary electrophoresis in agricultural and soil chemistry research In: He Z (ed) Capillary Electrophoresis: Fundamentals, Techniques and Applications. Nova Science Publishers, New York, p 131–151.

[26] He Z, Chapital DC (2015) Preparation and testing of plant seed meal-based wood adhesives. J. Vis. Exp. 97:e52557, doi: 52510.53791/52557 10.3791/52557 PMC4401199 25867092

[27] Pettigrew WT, Dowd MK (2011) Varying planting dates or irrigation regimes alters cottonseed composition. Crop Sci. 51:2155–2164.

[28] He Z, Senwo ZN, Zou H, Tazisong IA, Martens DA (2014) Amino compounds in poultry litter, litter-amended pasture soils and grass shoots. Pedosphere 24:178–185.

[29] He Z, Olk DC (2011) Manure amino compounds and their bioavailability In: He Z (ed) Environmental Chemistry of Animal Manure. Nova Science Publishers, Inc., N.Y., p 179–199.

[30] Perez PG, Zhang R, Wang X, Ye J, Huang D (2015) Characterization of the amino acid composition of soils under organic and conventional management after addition of different fertilizers. J. Soil. Sediments 15:890–901.

[31] Voet D, Voet J (1990) Chapter 4. Amino acids In: Biochemistry. John Wiley & Son, Inc., New York, N.Y., p 59–74.

[32] Martens DA, Frankenberger WT (1990) Determination of saccharides by high performance anion-exchange chromatography with pulsed amperometric detection. Chromatographia 29:7–12. 10.1016/s0021-9673(01)93027-4 1885699

[33] Martens DA, Loeffelmann KL (2002) Improved accounting of carbohydrate carbon from plants and soils. Soil Biol. Biochem. 34:1393–1399.

[34] Olk DC (2008) Improved analytical techniques for carbohydrates, amino compounds, and phenols: tools for understanding soil processes. Soil Sci. Soc. Am. J. 72:1672–1682.

[35] He Z, Shankle M, Zhang H, Way TR, Tewolde H, Uchimiya M (2013) Mineral composition of cottonseed is affected by fertilization management practices. Agron. J. 105:341–350.

[36] Berardi LC, Martinez WH, Fernandez CJ (1969) Cottonseed protein isolates: Two step extraction procedure. Food Technol. 23:75–82.

[37] Bodwell CE, Hopkins DT (1985) Nutritional characteristics of oilseed proteins In: Altschul AM, Wilcke HL (eds) New Protein Foods. Vol. 5. Seed Storage Proteins. Academic Press, New York, p 221–257.

[38] Bertrand JA, Sudduth TQ, Condon A, Jenkins TC, Calhoun MC (2005) Nutrient content of whole cottonseed. J. Dairy Sci. 88:1470–1477. 15778316 10.3168/jds.S0022-0302(05)72815-0

[39] Nida DL, Patzer S, Harvey P, Stipoanovic R, Wood R, Fuchs RL (1996) Glyphosate-tolerant cotton: the composition of the cottonseed is equivalent to that of conventional cottonseed. J. Agric. Food Chem. 44:1967–1974.

[40] Martinez WH, Berardi LC, Goldblatt LA (1970) Cottonseed protein products-composition and functionality. J. Agr. Food Chem. 18:961–968.

[41] King EE (1980) Compositional relationships among electrophoretic isolates from cottonseed protein bodies. Phytochem. 19:1647–1651.

[42] Hettiarachchy NS, Kalapathy U, Myers DJ (1995) Alkali-modified soy protein with improved adhesive and hydrophobic properties. J. Am. Oil Chem. Soc. 72:1461–1464.

[43] Wolfrum EJ, Lorenz AJ, deLeon N (2009) Correlating detergent fiber analysis and dietary fiber analysis data for corn stover collected by NIRS. Cellulose 16:577–585.

[44] Han YW (1988) Removal of phytic acid from soybean and cottonseed meals. J. Agric. Food Chem. 36:1181–1183.

